# Non-specific Symptoms of Small Bowel Neuroendocrine Tumor and the Diagnostic Challenges: A Case Report

**DOI:** 10.7759/cureus.41080

**Published:** 2023-06-28

**Authors:** Laith Daraghmeh, Sara Shbaita, Omar Nassef, Layan Melhem, Iyad Maqboul

**Affiliations:** 1 Department of General Surgery, An-Najah National University Hospital, Nablus, PSE; 2 Faculty of Medicine, An-Najah National University, Nablus, PSE; 3 Department of Radiology, An-Najah National University Hospital, Nablus, PSE; 4 Department of Pathology, An-Najah National University Hospital, Nablus, PSE; 5 Department of General Surgery, An-Najah National University Hospital, An-Najah National University, Nablus, PSE

**Keywords:** case report, abdominal pain, laparotomy, small bowel, neuroendocrine tumors

## Abstract

Neuroendocrine tumors (NETs) are considered the most frequent tumors that affect the small bowels. Despite diagnostic modalities, the diagnosis of small bowel NETs is not straightforward and poses a high challenge to most physicians, due to poor accessibility to this area and the patient's non-specific presentations.

We reported a case of a 60-year-old male patient, who presented with severe postprandial epigastric pain of one-month duration, loss of appetite, and weight loss. Investigations revealed no definite diagnosis. Therefore, exploratory laparoscopy was attempted along with multiple biopsies that led to the diagnosis of small bowel NET.

We conclude that NETs require a high index of suspicion in patients with recurrent abdominal pain.

## Introduction

Neuroendocrine tumors are slow-growing tumors that could occur in any of the body's organs. The gastrointestinal tract is considered the most vulnerable body site for neuroendocrine tumors, which are the foreground primary malignancy in the small bowel [1]. The incidence of NETs is 6.98 per 100000, which is 6.4-fold higher than in the 1970s [2]. This increase in NET incidence is explained by the new methods used in its diagnosis, and the better survival outcomes compared with adenocarcinoma [3].

The symptoms of NETs are related to the tumor site and whether it is functional or not, with the majority being non-functional, leading to no or very few symptoms [4]. The fact that the diagnosis of small bowel NETs is challenging, due to the wide variability in symptoms and presentations, intensifies the importance of being aware of it as a differential diagnosis in many scenarios.

Herein, we present a case in which a 60-year-old male patient presented with abdominal pain of one-month duration; throughout this duration, many investigations and medical management options were attempted, with neither improvement nor reaching a definite diagnosis. The decision was made to explore the abdomen laparoscopically, an area of suspected abnormality was resected in the small bowel, and biopsies were obtained to have the histopathology final definite diagnosis, which showed a small bowel NET.

## Case presentation

A 61-year-old male patient with no significant past medical and surgical history was referred to our hospital to continue investigations regarding his vague abdominal pain. The patient's history started 40 days prior to referral when he started to complain of postprandial epigastric abdominal pain that was gradual in onset, 20-30 minutes after each meal, colicky in nature, occasionally radiating to the flanks, with a severity of about 9/10 in some episodes according to his description. The pain was associated with loss of appetite, resulting in a weight loss of about 13 kilograms(kg) during the 40-day period. The patient mentioned a history of food aversion in an attempt to avoid exacerbation of his pain. He denied fever, nausea, vomiting, oral ulcers, odynophagia, dysphagia, changes in bowel movement, jaundice, chest pain, palpitation, dyspnea, orthopnea, paroxysmal nocturnal dyspnea, or cough. On admission, a thorough physical examination was unremarkable. Upon further questioning and reviewing the patient's records, his family history revealed that his father had died from lung cancer.

He initially sought medical help at an outpatient clinic and was prescribed over-the-counter unknown medications, but his symptoms didn't improve. A Helicobacter pylori (H. pylori) test was ordered from the same clinic when his symptoms didn't improve and returned positive, for which he was given triple therapy for H. pylori (esomeprazole, amoxicillin, and clarithromycin). The patient's symptoms didn't improve; accordingly, he was admitted to a governmental hospital one month before the current admission. Abdominal computed tomography (CT) scan without contrast upon admission was done and was unremarkable.

Gastroscopy followed and revealed findings of antral ulcer patches and a focal intestinal thickening. A biopsy was taken and showed chronic gastritis. This raised suspicion of untreated H. pylori infection, so treatment was resumed with triple therapy.

**Figure 1:**
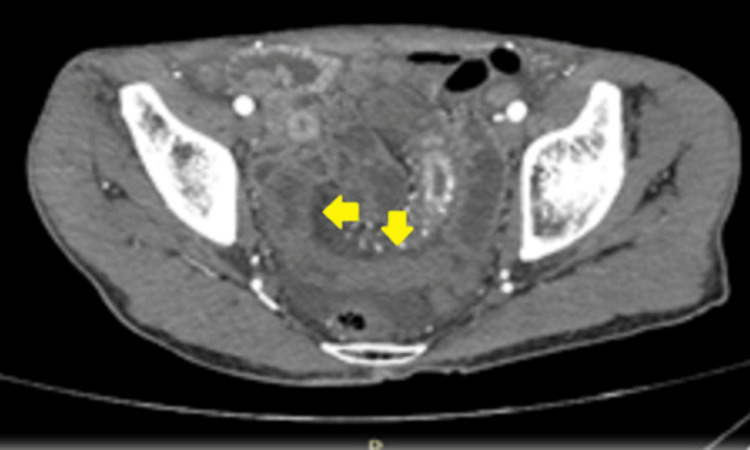
Abdomen and pelvic CT scan with IV contrast Imaging showed a circumferential wall thickening (yellow arrows) in a segment of the distal ileum

A few days later, the patient was discharged home. He was readmitted to the same hospital one week later due to increased abdominal pain frequency and severity. Another abdomen and pelvic CT scan with IV contrast showed new findings of ileal segment wall thickening, as well as a moderate amount of pelvic free fluid (Figure 1).

**Figure 2:**
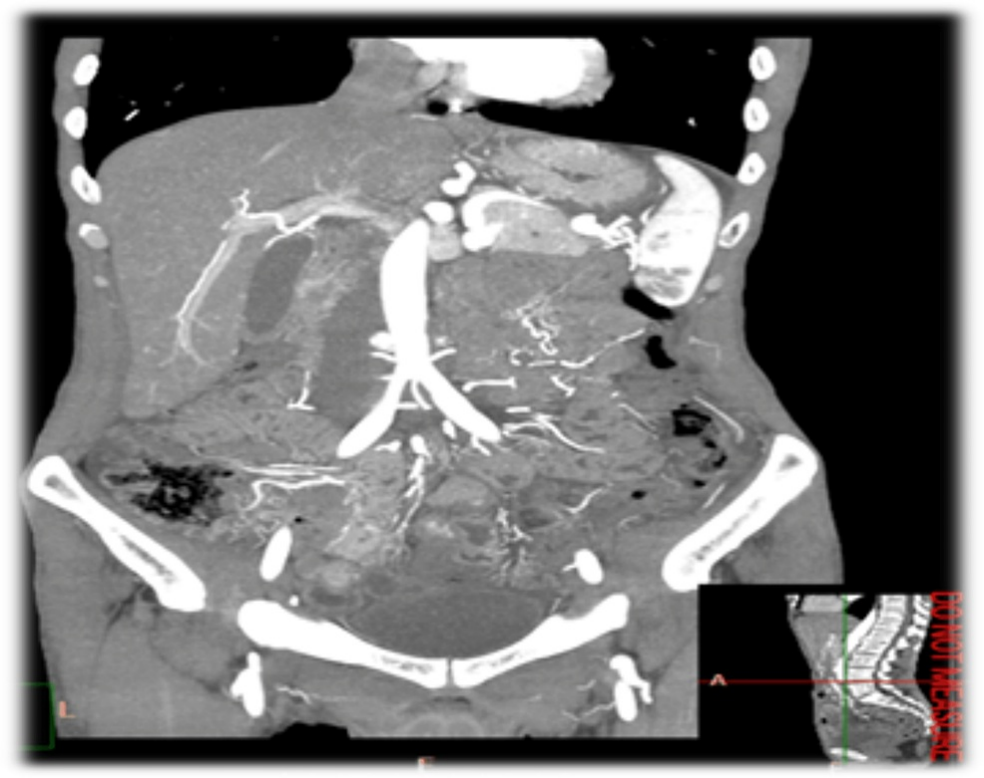
Abdominal CT angiogram Imaging showed patent abdominal vessels, normal angulation, and no signs of mesenteric artery obstruction

Hemorrhoids and two polyps in the transverse and descending colon were seen in a colonoscopy done at the same admission, along with a CT angiogram that was also unremarkable (Figure 2).

These investigations were inconclusive, and the need for further studies and a specialized hospital was mandatory. Accordingly, the patient was referred and admitted to our hospital for further evaluation.

Laboratory results upon admission are listed in Table 1. The stool analysis result was also normal.

**Table 1:** Initial laboratory results upon admission

Parameter	Result	Reference range
White blood cell count	5.5	4–9 k/µl
Hemoglobin	14.3	13.7–17.2 g/dL
Platelet	260	140–440 k/µL
Sodium	138	135–155 mEq/L
Potassium	3.32	3.5–4.8 mEq/L
Chloride	96.3	98–107 mEq/L
Blood urea nitrogen	7.2	5–22 mg/dL
Creatinine	0.68	0.7–1.2 mg/dL
Albumin	3.79	4–5 g/dL
C-reactive protein	35.1	0-5 mg/L
Erythrocyte sedimentation rate	35	0-15 mm/hr
Total bilirubin	0.449	0–1.2 mg/dL
Alkaline phosphatase	62	40–129 U/L
Gamma-glutamyl transferase	15	8-61 U/L
Aspartate transaminase	10.7	0–50 U/L
Alanine aminotransferase	7	13–59 U/L
Total protein	6.12	6.0–8.5 g/dL
Lactate	0.82	0-2 mmol/L

The patient’s carcinoembryonic antigen (CEA) was 2.19 ng/ml (reference range = ≤5.2 ng/mL), cancer antigen 19-9 (CA 19-9) was 9.77 (reference range = 0.0-39.0 U/mL), and alfa fetoprotein was 3.9 (reference range = ≤5.8 U/mL).

In the subsequent two days, the patient had multiple attacks of exacerbation of his pain, and analgesia was given along with esomeprazole 40 mg bid.

**Figure 3:**
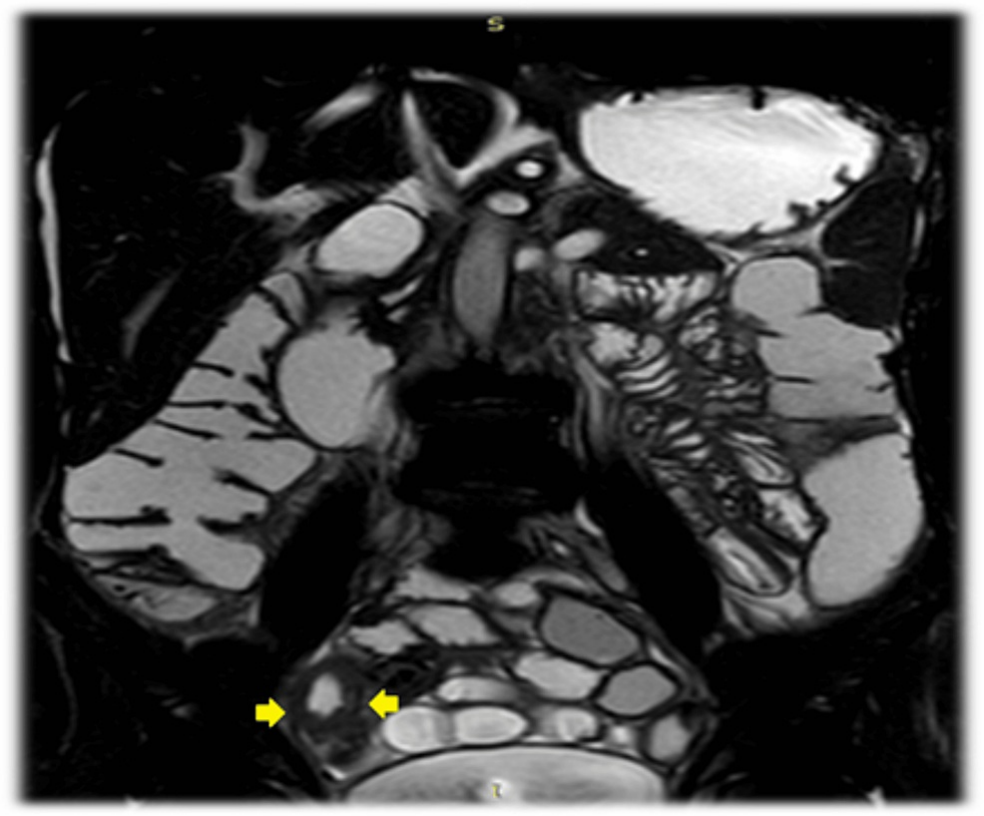
Small bowel enteroclysis magnetic resonance imaging (MRI) A coronal selected image of MR enteroclysis showing ileal wall thickening (yellow arrows) in the right lower abdominal quadrant

He was planned and prepared for small bowel enteroclysis magnetic resonant imaging (MRI) on the third day of admission, which revealed no bowel dilatation, stenosis, focal lesion, or any other findings other than the last CT scan (Figure 3). 

**Figure 4:**
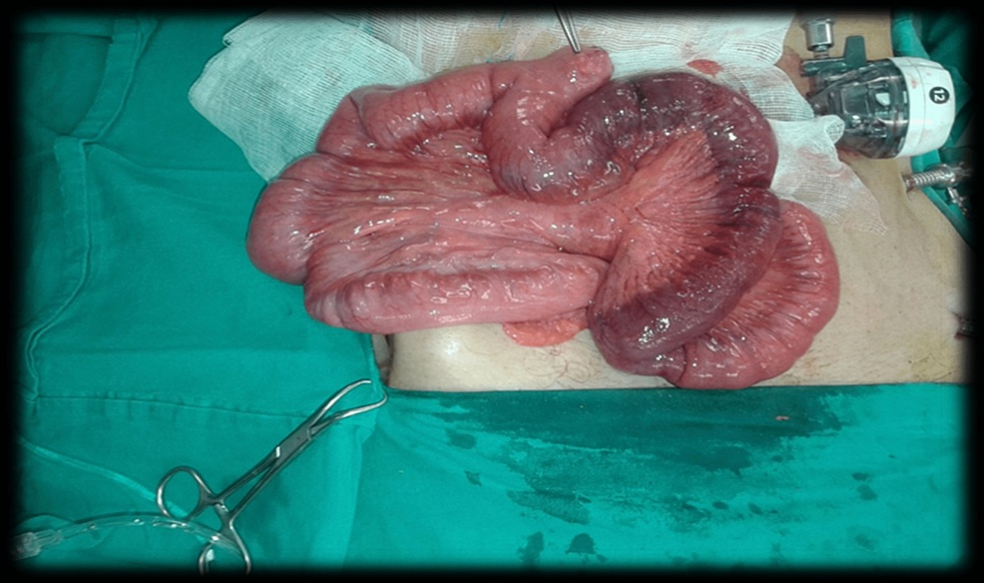
Intraoperative findings Abnormal part of the small bowel

The patient continued to complain of abdominal pain with an increase in its severity, so the decision was made to go for an exploratory laparoscopy with liver biopsy, omental biopsy, and free fluid aspiration. Intraoperatively, there was a suspicion of ischemia, and the question was if the appeared segment was viable or not, so the decision was to convert to open, in which exploration, the small intestine from the ileocecal valve to the duodenojejunal junction revealed the presence of multiple palpable masses about 1*2 cm each, 35 cm proximally away from the ileocecal valve. There was also an ischemic small bowel segment about 30 cm in length (70 cm from the ileocecal valve). According to these findings, the decision was to resect about 65 cm from the small bowel (32 cm proximal to the ileocecal valve) (Figure 4), with side-to-side anastomosis. Biopsies were taken as well and sent to histopathology.

**Figure 5:**
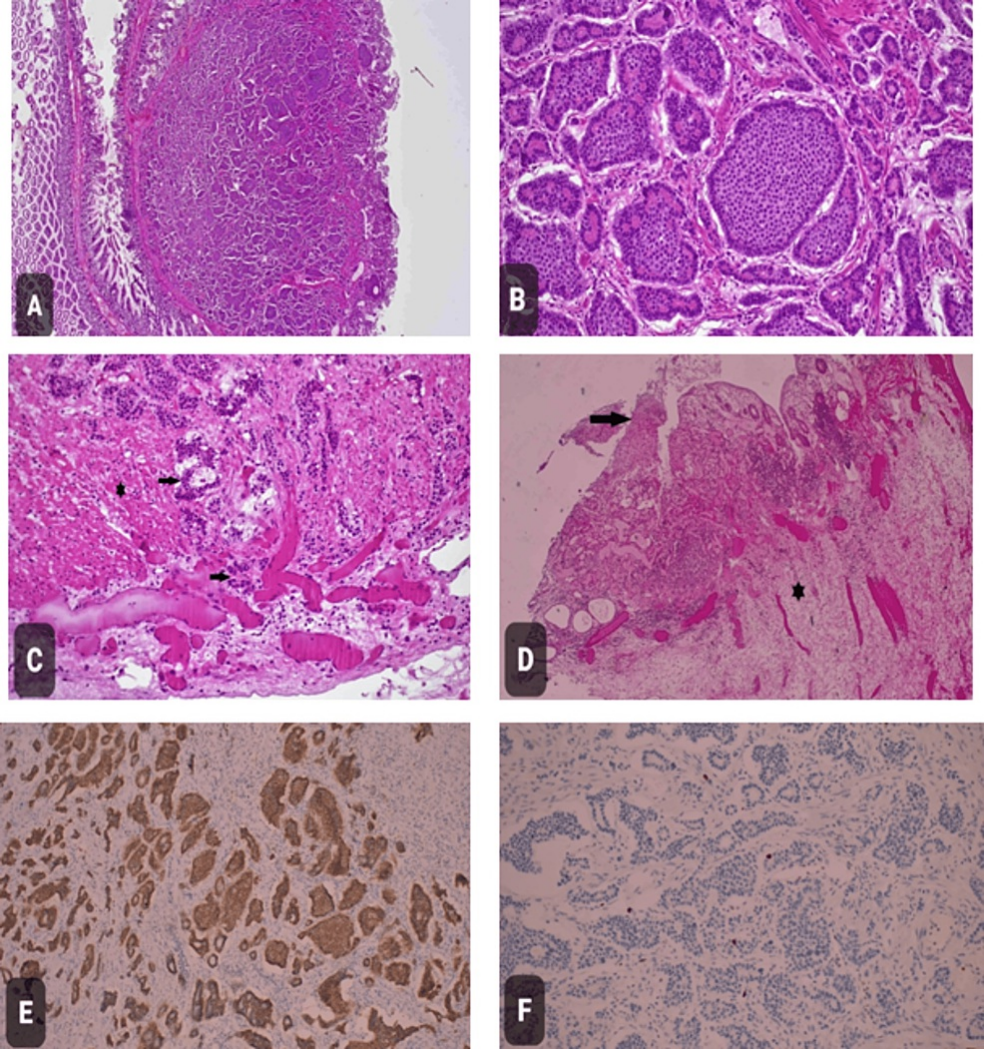
Histopathologic examination of the intraoperative samples A. Hematoxylin and eosin (H&E) staining of the ileal tumor (x20). B. H&E staining of the tumor (x200). C. H&E staining of tumor cells (black arrows) infiltrate through muscularis propria (asterisk) into subserosal tissue (x200). D. H&E staining of adjacent mucosa showing ischemic changes, including sloughing of the epithelial surface (black arrow) and submucosal congestion (asterisk) (x100). E. Tumor cells show diffuse expression of Synaptophysin immunostain (x200). F. The Ki-67 proliferation index is less than 3% (x200). Microscopic examination showed a neuroendocrine tumor (NET) with a classic nested pattern composed of monotonous cells with round nuclei and salt and pepper chromatin (A, B). Tumor cells infiltrated through the muscularis propria into subserosal tissue without penetration of the overlying serosa (C). The adjacent mucosa showed ischemic changes, including edema, congestion, and reactive inflammation along with focal sloughing of the epithelial surface (D). Tumor cells showed diffuse expression of synaptophysin (E). The Ki-67 proliferation index was less than 3% (F). The surgical resection margins were free of tumors. There were no lymph nodes resected with the specimen. A macroscopic examination of the resected small bowel segment showed a tumor measuring 1.3 cm in the greatest dimension. According to the WHO Classification 2019, the histologic grade for this tumor is a well-differentiated neuroendocrine tumor, G1. Given the tumor size and its extent, the pathologic stage classification (pTNM, AJCC 8th Edition) is pT3NxMX. pTNM: Pathological tumor-node-metastasis; AJCC: American Joint Committee on Cancer

The patient remains stable postoperatively. Meanwhile, histopathology showed a well-differentiated, grade 1 neuroendocrine tumor invading the muscularis mucosa into the subserosal tissue. All surgical margins were found to be uninvolved by the neoplasm (Figure 5). Lymph invasion wasn't identified. Furthermore, the liver biopsy showed liver cholestasis but no evidence of malignancy.

Before discharge, a staging chest CT scan showed no remarkable findings suggesting metastasis.

The patient was then discharged with recommendations of daily dressing over the wound, with instructions regarding follow-up with the oncology clinic. It is worth mentioning that the patient was lost to follow-up.

## Discussion

Our patient experienced a 40-day history of abdominal pain. Up to a quarter of the adult population will experience abdominal pain at any one time; most of these episodes resolve spontaneously. Abdominal pain can be acute or chronic, which is defined as lasting three months or longer [3]. There is a very wide differential diagnosis of abdominal pain. Understanding the pathophysiological principles of visceral pain and having knowledge of common gastrointestinal diseases and their natural history is very important for diagnosis. A careful history and physical examination with subsequent laboratory and imaging will generate clinical suspicion.

As about half of the patients older than 65 years who sought medical advice in the ED for abdominal pain are admitted, and as many as one-third require surgical intervention later on, abdominal pain must be considered serious [5]. The initial presentation, in this case, was to an outpatient clinic. He had a positive H. pylori test and triple therapy accordingly. However, this may be considered a drawback. Such patients may require admission for further evaluation or at least referral or further investigations in emergency departments to rule out life-threatening conditions; cardiac events are a good example of this situation.

Small bowel pain can be caused by motility disturbances and nonobstructing conditions, such as inflammation, ulcers, or neoplasms, which can be aggravated by food ingestion that may cause dull periumbilical or other midline discomforts [6]. Postprandial, colicky nature, and site of pain all are in favor of a small bowel origin of pain.

A neuroendocrine tumor is a term used to describe a uniquely wide variety of benign and malignant neoplasm subtypes that are mainly subdivided into poorly differentiated and well-differentiated, each characterized by features specified to the progenitor neuroendocrine cells they originate from. In addition, these cells have the ability to produce hormones and substances specific to each type [7].

NETs that produce active substances are called functional while those that do not are called nonfunctional. Forty percent (40%) of neuroendocrine tumors are found to be hormone producers, thus causing certain symptoms according to the hormone [8]. However, both types present with nonspecific symptoms initially, leading to misdiagnosis or late diagnosis and subsequently possible metastasis.

An international survey in 2014 that included 1928 patients with NETs revealed that most patients had delayed diagnosis, and 58% had metastasis at the time of diagnosis. Furthermore, patients reported many physician visits and extensive resources used to reach the diagnosis [8].

In surveillance of the Surveillance, Epidemiology, and End Results (SEER) database of 35,618 patients with NETs, the patients' age of onset was usually the sixth decade with a median of 63 years of age at the time of diagnosis [9].

In this case, the patient's age was 61 years old, and he presented with nonspecific symptoms of recurrent, severe abdominal pain, weight loss, and anorexia, and upon examination, no remarkable findings were found. This led to a delay in his diagnosis and many laboratory tests and imaging were done accordingly.

The most affected organs by NETs are the gastrointestinal (GI) tract and the bronchopulmonary systems. The GI tract constitutes what is about 55% of all cases, with the small intestine forming 45% [10]. The incidence of gastroenterology tract neuroendocrine tumors has risen since 1973 (1.5 cases per 1,000,000) to reach 4.6 cases per 1,000,000 in 2012 [11]. The increase in incidence in NETs cases generally and GI NETs cases are especially thought to be due to improving the diagnostic approaches and methods such as the use of gastroscopy, endoscopy, and other imaging modalities.

Small bowel neuroendocrine tumors (SBNETs) are now the most common types of tumors affecting the small intestine, and unlike adenocarcinomas, which mostly affect the duodenum, NETs are most likely to affect the ileum [3]. Smoking, family history of malignancy, and previous gallbladder disease are considered risk factors for developing SBNETs [12]. In our case, the patient had a family history of cancer, and his NET was found within the ileum, supporting previous data from different literature sources regarding the possible risk factors and the most vulnerable part of the small intestine.

Clinical symptoms of neuroendocrine tumors in the small bowels depend on their exact site and functionality. Most cases are nonfunctional and result in little to no symptoms in the early stage.

SBNETs exhibit symptoms if they metastasize to other organs or lymph nodes [13]. A large retrospective study by Zhang et al. found that 88.4% of all GI NETs from the study sample were nonfunctional and no symptoms have been documented [14]. In the early stages, around 20% of patients are diagnosed incidentally. On the other hand, the most common early symptom is abdominal pain, which promotes further investigations. Furthermore, in some cases of primary solitary tumors, extensive fibrosis with resultant narrowing, obstruction, and ischemia in the small bowel has been reported [15].

Late in the course, patients could have mass effect symptoms (bile duct obstruction causing jaundice, bowel obstruction, or palpable mass at examination) or metastasis-related symptoms. In metastasis, secreted hormones from the tumors will be able to bypass the portal circulation and so not be eliminated from the body; this will lead to carcinoid syndrome development (10% of all SBNET cases). The main symptoms of carcinoid syndrome are flushing, diarrhea, abdominal pain, heart valve involvement and possible destruction, edema, respiratory wheezing, and telangiectasia [16].

The patient in this case has been diagnosed with NETs 40 days following a vague abdominal pain. Metastasis and lymph node invasion were excluded with a follow-up CT scan after the diagnosis was made.

The SBNET diagnosis is considered challenging, with a high rate of misdiagnosis as a benign condition. This is due to the initially associated symptoms, which are mostly nonspecific as mentioned before, and their inaccessible site, which leads to late diagnosis and possible metastasis at the time of diagnosis. Metastasis at the time of SBNET diagnosis is 30%, and 40% will have lymph node invasion [1].

Diagnosis is achieved by a clinical approach accompanied by laboratory tests (useful in carcinoid syndrome cases to assess for hormone levels and other biochemical studies) and imaging, but histopathology of the obtained biopsies remains the confirmatory diagnostic method. However, the diagnostic approach should be directed to each case individually.

For localization and staging of small bowel NETs, both anatomic and functional imaging modalities are required; ultrasound (US), computed tomography (CT), and magnetic resonance imaging (MRI) are examples of anatomic modalities, whereas single-photon emission computed tomography (SPECT) and positron emission tomography (PET) describes functional ones. CT scan sensitivity for primary small bowel NETs ranges from 7% to 38%, but this can be improved to 82% if the presence of mesenteric lymphadenopathy or fibrosis is interpreted as evidence of a small bowel primary tumor [17].

Surgical resection is the standard of care in patients with locoregional disease [17]. Segmental small bowel resection with regional lymph node resection remains the optimal treatment option [18].

Histopathology retrospectively showed mucosal ischemic changes, as NETs may present with different clinical scenarios. In cases of mesenteric ischemia, the standard approach is CT angiography, which was done in our case and was unremarkable. There is a published case in 2017 of a midgut neuroendocrine tumor presented with acute intestinal ischemia. In that case, the patient complained of nonspecific symptoms for about one year before manifesting with acute mesenteric ischemia, and abdominal X-rays revealed pneumatosis intestinalis and an abdominal ultrasound and computed tomography confirmed the diagnosis. The patient was submitted to a segmental enterectomy [19].

Based on the European Society for Medical Oncology, follow-up investigations should include clinical symptom monitoring, biochemical parameters, and conventional and somatostatin receptor (SSTR) imaging. A second drawback in our case was that the patient was lost to follow-up, probably due to financial issues [20].

## Conclusions

Neuroendocrine tumors have a variety of clinical scenarios. These tumors should be kept in mind while dealing with patients with recurrent abdominal pain of unknown cause, requiring a high index of suspicion. Despite the advancement in imaging modalities, such tumors may not give clear clues of findings. Also, life-threatening causes of abdominal pain should be considered red flags and ruled out initially.
